# Benefits of a Pharmacology Antimalarial Reference Standard and Proficiency Testing Program Provided by the Worldwide Antimalarial Resistance Network (WWARN)

**DOI:** 10.1128/AAC.02362-14

**Published:** 2014-07

**Authors:** Chris Lourens, Niklas Lindegardh, Karen I. Barnes, Philippe J. Guerin, Carol H. Sibley, Nicholas J. White, Joel Tarning

**Affiliations:** a Worldwide Antimalarial Resistance Network (WWARN); b Mahidol-Oxford Tropical Medicine Research Unit, Faculty of Tropical Medicine, Mahidol University, Bangkok, Thailand; c Centre for Tropical Medicine, Nuffield Department of Medicine, University of Oxford, Oxford, United Kingdom; d Division of Clinical Pharmacology, Department of Medicine, University of Cape Town, Cape Town, South Africa; e Department of Genome Sciences, University of Washington, Seattle, Washington, USA

## Abstract

Comprehensive assessment of antimalarial drug resistance should include measurements of antimalarial blood or plasma concentrations in clinical trials and in individual assessments of treatment failure so that true resistance can be differentiated from inadequate drug exposure. Pharmacometric modeling is necessary to assess pharmacokinetic-pharmacodynamic relationships in different populations to optimize dosing. To accomplish both effectively and to allow comparison of data from different laboratories, it is essential that drug concentration measurement is accurate. Proficiency testing (PT) of laboratory procedures is necessary for verification of assay results. Within the Worldwide Antimalarial Resistance Network (WWARN), the goal of the quality assurance/quality control (QA/QC) program is to facilitate and sustain high-quality antimalarial assays. The QA/QC program consists of an international PT program for pharmacology laboratories and a reference material (RM) program for the provision of antimalarial drug standards, metabolites, and internal standards for laboratory use. The RM program currently distributes accurately weighed quantities of antimalarial drug standards, metabolites, and internal standards to 44 pharmacology, *in vitro*, and drug quality testing laboratories. The pharmacology PT program has sent samples to eight laboratories in four rounds of testing. WWARN technical experts have provided advice for correcting identified problems to improve performance of subsequent analysis and ultimately improved the quality of data. Many participants have demonstrated substantial improvements over subsequent rounds of PT. The WWARN QA/QC program has improved the quality and value of antimalarial drug measurement in laboratories globally. It is a model that has potential to be applied to strengthening laboratories more widely and improving the therapeutics of other infectious diseases.

## INTRODUCTION

Accurate blood or plasma drug concentration measurement is an essential component of the assessment of therapeutic failure so that true resistance can be differentiated from inadequate drug exposure. When pharmacokinetic data are assessed, it is necessary to differentiate between true patient population differences and differences in accuracy between assay methods or laboratories.

Six Worldwide Antimalarial Resistance Network (WWARN) scientific groups, (Pharmacology, *In Vitro*, Molecular, Clinical, Antimalarial Quality, and Informatics) located in centers of excellence around the world, offer a platform for collaborative research into the understanding, identification, and spread of antimalarial drug resistance. Each group specializes in different aspects of antimalarial drug resistance and works collaboratively to develop customized tools and services designed to facilitate quality-assured data collection, analysis, and reporting. The WWARN laboratory quality assurance/quality control (QA/QC) program (1) provides access to high-quality reference standards, eliminates weighing errors, and supports pharmacology, *in vitro*, and drug quality testing laboratories, including those in low-resource settings, to achieve high-quality data.

An understanding of the relationship between antimalarial drug exposure and therapeutic efficacy is essential for informing optimal dosing in key target populations. Pharmacokinetic-pharmacodynamic (PK-PD) relationships have not been well characterized for most of the antimalarial drugs in current use, which limits the evidence base needed for prolonging their useful therapeutic life (2). Most PK-PD studies of antimalarial drugs are not powered adequately to define these relationships, necessitating pooling of individual patient data from as many studies as possible. Individual patient data pooling requires that there are no significant systematic differences between laboratories in the accuracy of drug concentration measurements. Cross-validation of analytical laboratories shows variability in pharmacological data between laboratories, which includes both systematic and random between-laboratory errors that might wrongly be attributed to population differences if not accounted for (3). This report describes the WWARN antimalarial drug QA/QC program operation and performance.

## MATERIALS AND METHODS

### Proficiency testing.

The WWARN QA/QC proficiency testing program for pharmacology laboratories assesses the ability of pharmacology laboratories to assay blood or plasma samples for concentrations of antimalarial compounds and their metabolites. Participation in the proficiency testing program is open to all laboratories doing either therapeutic efficacy studies or other research on antimalarial drug exposure. The program currently offers plasma-based samples for eight antimalarial drug compounds and metabolites: chloroquine/desethylchloroquine, mefloquine/carboxymefloquine, primaquine/carboxyprimaquine, amodiaquine/desethylamodiaquine, piperaquine, lumefantrine/desbutyl-lumefantrine, dihydroartemisinin, and artesunate. Commercially obtained and controlled plasma is spiked with accurately weighed certified reference materials. All active ingredients and the plasma are controlled by the manufacturer and reflected in certificates of analysis. Each analyte is sent in a range of concentrations, including the highest and lowest concentrations expected to be found in clinical samples (Table 1), which allows each laboratory to test the limits of its assay.

**TABLE 1:** Proficiency testing of plasma samples spiked with certified antimalarial compounds and metabolites to allow each laboratory to test the limits of its assay

Analyte	Concn (ng/ml)	
	Low	High
Amodiaquine	4.0	4,192
Desethylamodiaquine	4.0	4,192
Lumefantrine	25	20,000
Chloroquine	4.0	4,192
Desethylchloroquine	4.0	4,192
Dihydroartemisinin	1.6	2,875
Piperaquine	1.2	575
Mefloquine	70	4,000
Carboxymefloquine	70	4,000
Primaquine	10	400
Carboxyprimaquine	50	4,000

### Completed proficiency testing.

Each round of proficiency tests consisted of three cycles per year, starting approximately in July and ending in June of the following year. At the end of every cycle of 4 months, the anonymized results from participating laboratories were evaluated against the nominal concentration (assigned value) for each antimalarial drug/metabolite. The performance of the pharmacology laboratories that are currently enrolled in the program was assessed and evaluated in terms of their ability to assay the supplied spiked antimalarial plasma samples during four rounds of proficiency testing between 2010 and 2013. Performance is reported using *Z*-scores (4), calculated according to the following equation: *Z* = (*x* − *x_a_*)/SDPA, where *x* is the participant result, *x_a_* is the nominal value, and SDPA is the standard deviation for proficiency assessment. The revised Harmonized Protocol (5) describes several ways in which the SDPA can be obtained. It can, for example, be determined in a proficiency test as the standard deviation of all the laboratory results (excluding significant outliers). However, the Harmonized Protocol recommends that SDPA be a set value which corresponds to the precision needed to perform a certain task. The Harmonized Protocol calls this SDPA the “fitness-for-purpose-based” standard deviation for proficiency assessment. The WWARN QA/QC unit calculates the SDPA for each QC sample based on the lower limit of quantification (LLOQ) reported by the laboratory (1). This means that the same QC sample could produce different SDPAs for different laboratories depending on the LLOQs for their respective methods. The absolute *Z*-scores (|*Z*|) are evaluated according to the following criteria: |*Z*| ≤ 2, the result is considered satisfactory; 2 < |*Z*| ≤ 3, the result is considered questionable; |*Z*| ≥ 3, the result is considered unsatisfactory.

### Reference material program.

The WWARN QA/QC laboratory issues small, accurately weighed samples of a large number of reference materials (http://www.wwarn.org/toolkit/qaqc/reference-material-scheme) to research groups that are members of the network at no cost. The WWARN QA/QC reference material program applies principles of good weighing practice (6) by using a Sartorius SE2 Ultra microbalance (Sartorius AG, Germany). Antimalarial standards and common metabolites are provided in 20-mg quantities in either glass or polypropylene vials. The receiving laboratories then use their own procedures of weighing accurate quantities to prepare stock and working standard solutions. A laboratory may also request quantities of 0.5 mg to 1 mg, to which an appropriate amount of solute can be added to produce reference stock solutions (RSS) ranging between 0.5 and 1 mg/ml.

## RESULTS

### Proficiency testing.

**FIG 1:**
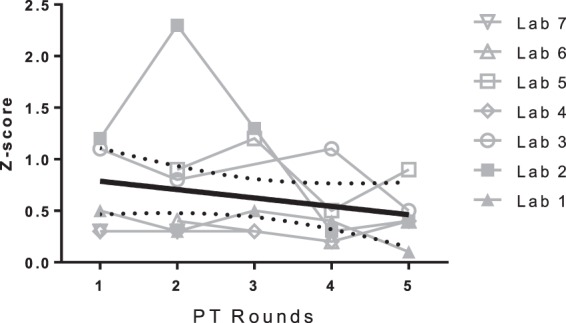
Overall *Z*-scores over five rounds of proficiency testing (PT). The solid black line is a linear regression of all available *Z*-scores, with broken black lines indicating the 95% confidence interval. Results were scored as follows: |*Z*| ≤ 2, satisfactory; 2 < |*Z*| ≤ 3, questionable; and |*Z*| ≥ 3, unsatisfactory. Only seven laboratories analyzed this particular antimalarial compound (i.e., chloroquine).

A total of eight laboratories from six countries on five continents participated in at least three rounds of proficiency testing during the evaluation period. All laboratories used established extraction methods followed by liquid chromatography-mass spectrometry (LC-MS) or LC-UV detection. Laboratories were free to select their preferred assay method in order to have their performance assessed in the proficiency testing program. When performance scores over several rounds of proficiency testing for all laboratories and a particular antimalarial compound (7) were combined, it was evident that there was an overall improvement over time, as indicated with a downward trend in *Z*-scores (Fig. 1). A linear regression of all available *Z*-scores decreased the fitted value of the regression line from 0.79 to 0.46 between the first and last round of proficiency testing, indicating a 41% improvement in overall results. Some laboratories returned consistently high quality results when measured against external controls. In other laboratories, the WWARN QA/QC proficiency testing program was able to point out several discrepancies and advised laboratories on the examination of both random errors and systematic errors as possible root causes of any unsuccessful assay performance.

Random errors arise as a result of chance variations in factors that influence the value of the drug and metabolite concentration being measured. For example, electrical noise, thermal effects, or chance contaminations that are normally outside the control of an analyst may cause results to vary in an unpredictable way (8). These errors are not easy to control, but the results of random errors are usually easy to identify and can be treated as outliers in the analysis of the results. Systematic errors remain constant or vary in a predictable way over a series of measurements. Systematic errors can be corrected if they are detected.

**FIG 2:**
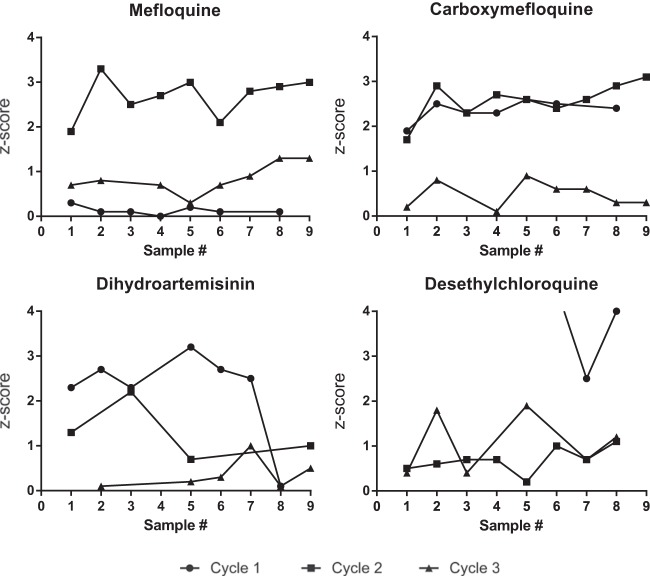
Example of performance over three cycles of proficiency testing in a particular laboratory for four antimalarial compounds assayed. Results were scored as follows: |*Z*| ≤ 2, satisfactory; 2 < |*Z*| ≤ 3, questionable; and |*Z*| ≥ 3, unsatisfactory. Sample numbers refer to individual plasma samples per cycle.

Figure 2 illustrates the influence of the proficiency testing program in a sample laboratory that adjusted its methods in response to technical advice given, showing consistently improving performance between the first and last cycle. Examples of unsuccessful performance detected by the proficiency testing program included systematic biases in preparation of stock solutions and working solutions, incorrect formulations (e.g., phosphate/base formulation and impurity issues), incorrect dilution schemes, and wrongly reported units of concentration.

### Reference material program.

**FIG 3:**
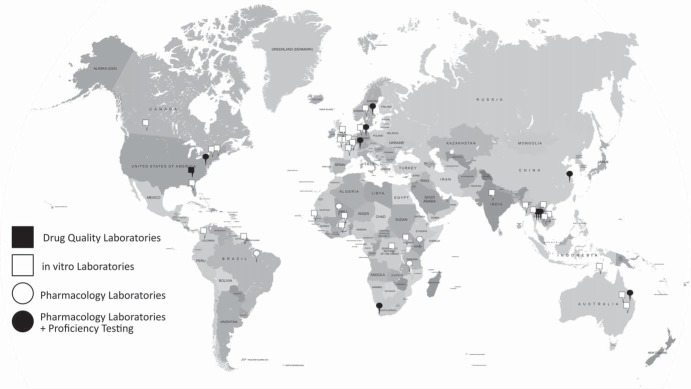
Distribution map of pharmacology proficiency testing laboratories, *in vitro* laboratories, and drug quality testing laboratories (updated December 2013). All laboratories received reference materials.

Antimalarial reference standards, metabolites, and internal standards are currently distributed to 14 pharmacology laboratories and 30 *in vitro* laboratories across six continents (Fig. 3). Eight of these 14 pharmacology laboratories participate in the proficiency testing program. Only one laboratory testing antimalarial drug quality requested reference materials. By utilizing the same source of standards and same accurate weighing procedures for all laboratories, bias arising from poor-quality standards and weighing inaccuracies is minimized.

Each reference standard is accurately weighed, and the balance has a measured uncertainty of ±0.0013 mg at the 1-mg level, resulting in a maximum of 0.13% relative error at this level (9).

**FIG 4:**
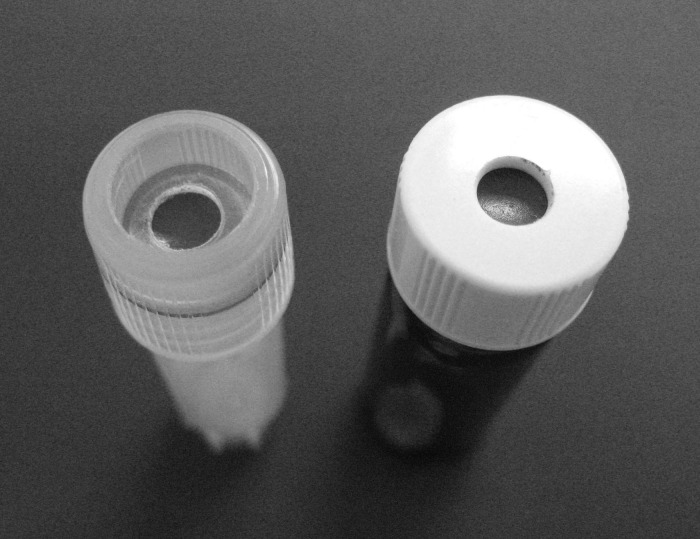
Reference material vials with pierceable silicone/Teflon septa.

For internal standards and some of the rarer metabolites, amounts of 0.5000 to 1.0000 mg are accurately weighed using an inert disposable microweighing boat. When an amount of 0.5000 mg is weighed, it should introduce a maximum relative error of ±0.26%. The precise weight is recorded, and the weighing boat is transferred to a 2-ml cryovial. Pairs of cryovials are sent to requesting laboratories where an appropriate amount of solute can be added to produce RSS. Vials have an opening in the cap with a pierceable silicone/Teflon septum (Fig. 4). The vial will therefore not be opened prior to preparation of stock solution in order to minimize the risk for possible loss of compound due to differences in air pressure and/or static electricity.

## DISCUSSION

A substantial improvement in antimalarial drug measurement performance was shown over the course of testing for nearly all participating laboratories, highlighting the advantage of an external quality assessment program. While two participating laboratories performed to a satisfactory degree from the start, the rest have demonstrated substantial improvements over subsequent rounds of proficiency testing. Results presented here showed a 41% reduction in overall *Z*-scores from the first round to the last round of proficiency testing. This trend most likely reflected laboratories optimizing their methods, paying more attention to quality control, and performing antimalarial drug assays to higher standards. Participating laboratories have benefited from this external monitoring of their performance of several antimalarial drug assays and from technical advice to correct any discrepancies identified. These were usually easily rectified by applying systematic root cause analysis to discover the reason(s) for systematic errors, and when corrective measures were applied, more accurate results were observed for subsequent analyses. An additional advantage for laboratories participating in the proficiency testing program is that participants may communicate their own results, including the regular program reports, privately to a laboratory accreditation or other assessment body, when required for the purpose of assessment, or to clients for the purpose of demonstrating analytical capability.

Even in good laboratory practice (GLP)/accredited industry laboratories, it is not uncommon for internal QC samples to fail to mimic clinical study samples or externally prepared QC samples (10). Once the method has been fully validated and meets internal acceptance criteria for QCs (11), discrepancies are usually caused and/or influenced by a number of factors, as follows.

### (i) Systematic differences in the background matrix.

External QC samples will have a different background composition (e.g., salts, proteins, lipids, and disease markers) than the in-house blank matrix used for preparation of calibration standards and QC samples. It is crucial to test a variety of different matrix sources during validation to evaluate these matrix effects (i.e., how changes in the background composition influence the precision and accuracy of the method).

### (ii) Improper internal standard.

The ideal method with mass spectrometric detection is to use a stable-isotope-labeled internal standard. Identical structures means that this should theoretically compensate for any variation during sample preparation, e.g., increased salt content in study samples that could decrease recovery on a weak ion exchanger solid-phase extraction column. A stable-isotope-labeled internal standard has the same affinity for the active sites as the analyte and would therefore compensate for this decreased recovery. An analogue internal standard might fail to compensate because it might have higher/lower affinity for active sites than the drug of interest. An isotope-labeled internal standard should theoretically also compensate for matrix effects (see paragraph i above) since it has identical retention times on the liquid chromatography column as the analyte. However, small differences in retention times might be seen between an isotope-labeled internal standard and an analyte which in rare cases fail to compensate for matrix differences (12).

### (iii) Carryover.

Calibration standards and QC samples are often analyzed in order of increasing analyte concentration. The drug and metabolite concentrations of study samples or external QC samples are not known, and carryover may become a problem.

### (iv) Systematic bias in preparation of stock solution and working solutions.

Incorrect assumptions about the formulation of standards used (e.g., phosphate/base formulation, impurity, and/or water content) or dilution schemes can create a systematic bias in the calibration samples and QC samples that might be difficult to identify.

### (v) Reported units of concentrations.

Not all studies utilize the same units of concentration when reporting results, and it is the responsibility of the proficiency testing provider to specify what units are to be used to evaluate submitted results.

To evaluate the potential causes for differences between actual drug and/or metabolite concentrations measured and the nominal values, the following steps can be performed fairly quickly.

(i) A simple way to evaluate for systematic differences in the background matrix or an improper/suboptimal internal standard is to rerun external QC samples, both naive and also with 1:5 and 1:10 dilutions using the laboratory internal blank source. Matrix effects and/or recovery differences are minimized. If these dilutions give a result significantly different from the nondiluted sample, then the method suffers from a problem of matrix and/or recovery effects, and the internal standard fails to compensate effectively for these.

(ii) Carryover can also be easily excluded by analyzing a batch of extracted blank samples and zero samples randomly allocated within a batch. Carryover does not always arise in the autosampler but could also come from steps during sample preparation. This experiment evaluates the whole method procedure.

(iii) Systematic bias in preparation of stock and working solutions may be identified through careful review of preparation schemes and proper traceability of performed preparations, assessing whether all calculations and the factors for formulation and purity are correct. Some laboratories implement two different stock/working solution sets, one for standards and one for QC samples, which helps to detect if one preparation should fail.

(iv) Inaccuracies with the reported units of concentrations are avoided by issuing a standardized result form to all participants. Test results should be converted to comply with the desired units of the result form.

The value of the WWARN QA/QC reference material program is evident when the program is evaluated against the cost and availability of antimalarial reference compounds, metabolites, and internal standards. Purchased individually by independent laboratories, the cost of small quantities of these compounds might be prohibitive, and the quality from small manufacturers might be questionable. The WWARN QA/QC unit negotiated a very favorable price by acquiring larger quantities, with the added advantage of a memorandum of understanding between WWARN and the manufacturer to retest the compounds close to expiry dates for recertification. This recertification process was included in the original price for the compounds. Any compounds not readily available from the manufacturer are custom synthesized and are provided with a comprehensive certificate of analysis. The certificates are distributed and updated to all participants of the WWARN QA/QC program. The potential for standardization across laboratories is now also possible since all participating laboratories have the opportunity to make use of the same reference standards for calculating a standard curve and internal QC samples. The reference material program is highly regarded by pharmacology laboratories, and the program has also distributed reference materials to *in vitro* laboratories to assess whether interlaboratory variability of *in vitro* drug susceptibility testing could be minimized by introducing simple standardization measures. The results of this study will be used to design a proficiency testing program to improve standardization of *in vitro* assessment across the malaria community. The WWARN QA/QC reference material program in collaboration with the WWARN Antimalarial Quality scientific group will provide antimalarial medicine standards, metabolites, and internal standards to bioanalytical laboratories testing for drug quality. The QA/QC unit may also develop a proficiency test program for these laboratories specifically.

### Conclusion.

The WWARN QA/QC program has demonstrated its potential for facilitating quality-assured antimalarial drug and metabolite pharmacokinetics and *in vitro* assays. It is evident that similar programs might benefit researchers seeking to improve the assays used in evaluating other antimicrobials (13).
